# Low‐Density Lipoprotein Cholesterol, Cardiovascular Risk Factors, and Predicted Risk in Young Adults

**DOI:** 10.1002/clc.70009

**Published:** 2024-09-09

**Authors:** Alexander R. Zheutlin, Samuel Luebbe, Alexander Chaitoff, Eric L. Stulberg, John T. Wilkins

**Affiliations:** ^1^ Division of Cardiology Feinberg School of Medicine, Northwestern University Chicago Illinois USA; ^2^ Department of Medicine University of Michigan Ann Arbor Michigan USA; ^3^ Department of Neurology University of Pennsylvania Philadelphia Pennsylvania USA

**Keywords:** cardiovascular risk, low‐density lipoprotein cholesterol, young adults

## Abstract

**Background:**

Young adults with elevated LDL‐C may experience increased burden of additional cardiovascular disease (CVD) risk factors. It is unclear how much LDL‐C levels, a modifiable factor, correlate with non‐LDL‐C CVD risk factors among young adults or how strongly these CVD risk factors are associated with long‐term predicted CVD risk. We quantified clustering of non‐LDL‐C CVD risk factors by LDL‐C among young adults to assess the association between non‐LDL‐C and LDL‐C risk factors with predicted CVD risk in young adults.

**Methods:**

The current analysis is a cross‐sectional study of adults < 40 years with an LDL‐C< 190 mg/dL participating in the National Health and Nutrition Examination Survey (NHANES) between January 2015 and March 2020. We measured the prevalence of non‐LDL‐C risk factors by LDL‐C and association between LDL‐C and non‐LDL‐C risk factors with predicted risk of CVD by the Predicting Risk of cardiovascular disease EVENTs (PREVENT) equations.

**Results:**

Among 2108 young adults, the prevalence of LDL‐C ≥ 130 mg/dL was 15.5%. Compared with young adults with LDL‐C < 100 mg/dL, those with LDL‐C 100–< 130, 130–< 160, and 160–< 190 mg/dL had greater non‐LDL‐C risk factors. Both LDL‐C and non‐LDL‐C risk factors were independently associated with a 30‐year risk of CVD (OR 1.05, 95% CI 1.03–1.07 and OR 1.17, 95% CI 1.12–1.23, respectively). The association of LDL‐C and 30‐year risk did not vary by non‐LDL‐C risk factor burden (*p*_interaction_ = 0.43).

**Conclusion:**

Non‐LDL‐C risk factors cluster among increasing levels of LDL‐C in young adults. Greater guidance on how to manage cardiovascular risk factors in young adults is needed.

## Graphical abstract

We assessed associations between low‐density lipoprotein cholesterol (LDL‐C) levels and non‐lipid CVD risk factors as well as 10‐ and 30‐year risk of CVD in young adults (< 40 years) participating in the nationally representative sample National Health and Nutrition Examination Survey. Many young adults with an elevated LDL‐C have concomitant high‐risk CVD characteristics, as well as long‐term CVD risk. The risk/benefit calculous for addressing this modifiable risk factor in these patients should be further explored.

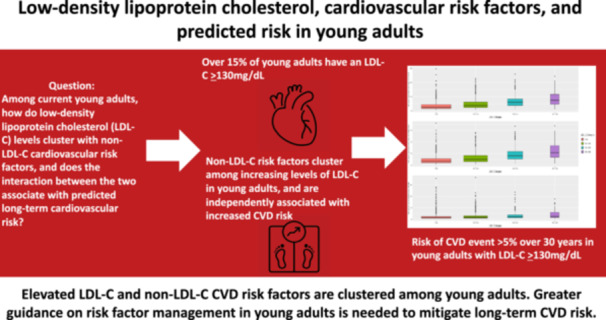

**Abbreviations**
ASCVD: atherosclerotic cardiovascular disease
BMI: body mass index
BP: blood pressure
CAD: coronary artery disease
CKD: chronic kidney disease
CVD: cardiovascular disease
HDL‐C: high‐density lipoprotein cholesterol
HF: heart failure
LDL‐C: low‐density lipoprotein cholesterol
non‐LDL‐C: non‐low‐density lipoprotein cholesterol
PCE: Pooled Cohort Equation
PREVENT: predicting risk of cardiovascular disease events

## 1 Introduction

Despite advances in the management of cardiovascular disease (CVD), optimal management strategies of cardiovascular risk factors in young adults remain elusive. Young adults in the United States face a growing burden of CVD risk factors, including elevated low‐density lipoprotein cholesterol (LDL‐C), blood pressure (BP), obesity, and insulin resistance [1, 2]. Atherogenic lipids are a readily modifiable risk factor for CVD with strong evidence to suggest that early life burden is associated with later life risk for CVD [3, 4].

Though the burden of CVD risk factors is high among young adults, it is unclear how atherogenic lipid levels cluster with other risk factors among young adults in the United States. Characterizing the overlap among lipid and non‐lipid risk factors may be helpful to inform clinical strategies specific to cardiovascular risk reduction in young adults. To reduce CVD‐related morbidity and mortality in mid‐ and late life, clinical practice guidelines need to incorporate how to monitor and manage lipid and non‐lipid risk factors in young adults—particularly given the interconnectedness between atherogenic lipids and non‐lipid CVD risk factors, as well as CVD risk across the lifespan. Determining the cumulative burden of clustered CVD risk factors among young adults may help to inform policy and clinical practice guidelines to individually and collectively address these risk factors among young adults—a population that may see the largest benefit with appropriate risk reduction over time.

To address these questions, we used the National Health and Nutrition Examination Survey (NHANES), which offers nationally representative data of young adults (≥ 18 and < 40 years of age) in the United States. We first characterized the clustering of non‐LDL‐C cardiovascular risk factors by LDL‐C levels among young adults. Second, we described of 10‐ and 30‐year predicted risk of CVD, atherosclerotic cardiovascular disease (ASCVD), and heart failure (HF) among young adults as Predicting Risk of cardiovascular disease EVENTs (PREVENT) by LDL‐C and their association with LDL‐C and non‐LDL‐C risk factors, separately [5, 6].

## 2 Methods

### 2.1 Study Design and Population

We extracted data on individuals aged ≥ 18 and < 40 years from NHANES between January 2015 and March 2020. The NHANES survey captures the health status of civilian, noninstitutionalized members of the US population creating a nationally representative multistage sampled cohort [7]. NHANES uses trained interviewers to administer standardized questionnaires in participants’ homes. Following the interview, participants are invited to mobile examination units for clinical data collection, including examination metrics as well as urine and blood samples. The NHANES protocol is approved by the National Center for Health Statistics Institutional Review Board and all participants provided informed consent.

We included respondents sampled to be a part of the fasting laboratories subgroup, which contains the LDL‐C variable, as calculated by the Friedewald equation, as well as data on our covariates of interest. We excluded adults with potential familial hypercholesterolemia (defined as having an LDL‐C > 190 mg/dL) who would have a separate indication for LLT (*n* = 27), those with a history of coronary heart disease (*n* = 3), or those already on LLT (*n* = 24). Of the 5486 NHANES participants ≥ 18 years old and < 40 years during the study period, 54 were excluded for above stated conditions, an additional 3324 were excluded for not being part of the fasting laboratories subgroup, yielding a final analytic sample of 2108 individuals.

### 2.2 Non‐LDL‐C Risk Factors

We obtained respondents’ interview responses to demographic characteristics (self‐reported age, sex, and race/ethnicity), health behaviors (self‐reported smoking status), medications (self‐report with interviewer pill bottle review), and personal or family history of cardiometabolic medication conditions (self‐reported). We extracted examination results including blood pressure (BP), body mass index (BMI), and waist circumference. Finally, we collected data on laboratory results, including triglycerides, high‐density lipoprotein‐cholesterol (HDL‐C), LDL‐C, glycosylated hemoglobin (HbA1c), fasting glucose, high‐sensitivity C‐reactive protein (hs‐CRP), and serum creatinine.

Risk factor definitions were informed by the 2018 ACC/AHA Management of Blood Cholesterol Guidelines [8]. Respondents were considered to have a family history of premature ASCVD if they reported they had a first‐degree relative with a heart attack or angina that began at < 50 years of age. Respondents were considered to have hypertension if they met any of three criteria: (1) systolic BP (SBP) > 130 mmHg or diastolic BP (DBP) > 80 mmHg, (2) self‐reported diagnosis of hypertension, or (3) antihypertensive medication use. We defined a respondent as having metabolic syndrome if three or more of the following criteria were met: waist circumference of > 40 inches in men and > 35 inches in women, triglycerides ≥ 150 mg/dL, HDL‐C < 40 mg/dL in men and < 50 mg/dL in women, BP ≥ 130/80 mmHg or taking antihypertensive medication, fasting glucose of 100 mg/dL or taking antidiabetic medication [9]. Respondents were considered to have chronic kidney disease (CKD) if they met one of two criteria: (1) estimated glomerular filtration rate (eGFR) < 60 mL/min/1.73 m^2^ using the CKD‐EPI equation or (2) self‐reported diagnosis of CKD [10]. Respondents were considered to have diabetes if they met any of four criteria: (1) fasting glucose ≥ 126 mg/dL, (2) HbA1c ≥ 6.5%, (3) self‐reported diabetes mellitus, or (4) antidiabetic medication use. Finally, we used specific laboratory cutoffs, as defined by the 2018 ACC/AHA Management of Blood Cholesterol Guidelines, to indicate potential risk‐enhancing factors [8]. These included elevated fasting blood glucose (> 100 mg/dL), low HDL‐C (< 40 mg/dL in males, < 50 mg/dL in females), triglycerides (≥ 175 mg/dL), and hs‐CRP (≥ 2 mg/L). For ease of interpretation all the above factors are designated as non‐LDL‐C CVD risk factors.

### 2.3 PREVENT Calculators

To calculate 10‐ and 30‐year risk of cardiovascular events, we used the base AHA PREVENT calculator [5, 6]. Briefly, the PREVENT calculator was derived from individual‐level data from over 3 000 000 individuals from 25 data sets and externally validated in over 3 000 000 individuals from 21 unique data sets [5, 6]. This model improves upon the determination of cardiovascular events from the Pooled Cohort Equations (PCEs) and is validated for adults as young as 30 years of age [5, 6]. The outcomes for which risk can be predicted include ASCVD and HF, as well as CVD, which is a combination of ASCVD and HF [5, 6]. We used the base PREVENT equation to calculate 10‐ and 30‐year risk to allow for the broadest inclusion of young adults with clinically available inputs. Given that our sample had young adults below the threshold of 30 years of age, for participants < 30 years of age, an age of 30 years was imputed to provide the lowest validated age in the PREVENT risk calculator.

### 2.4 Statistical Analysis

NHANES‐provided sample weights were used to account for the complex survey design and ensure a nationally representative analytic sample. Taylor‐series linearization was used to estimate sampling errors [7]. Following weighting, we described the sample characteristics stratified by level of LDL‐C representing varying levels of elevation above optimal (< 100 vs. 100–< 130, 130–< 160, 160–< 190 mg/dL) [11]. We also reported LDL‐C‐stratified prevalence estimates of non‐LDL risk factors as well as predicted 10‐ and 30‐ year CVD, ASCVD, and HF risk using PREVENT equations.

To understand the association between LDL‐C with non‐LDL‐C risk factor burden, we used bivariate logistic regressions to evaluate the associations between ordinal LDL‐C level with each non‐LDL‐C risk factor. To determine the association between LDL‐C and non‐LDL‐C risk factors on predicted 30‐year risk of CVD, ASCVD, and HF, we performed beta regressions with sequential models accounting for confounding and effect modification. Model 1 was unadjusted. Model 2 was adjusted for age, gender, race, LDL‐C, cumulative non‐LDL‐C risk factors (metabolic syndrome, enlarged waist, hypertension, elevated triglycerides, low HDL‐C, elevated glucose, elevated hs‐CRP, CKD). Model 3 was adjusted for all variables in Model 2 with the addition of an (LDL‐C)*(number of non‐LDL risk factors) interaction term. All characteristics were presented as weighted prevalence‐estimates with 95% confidence intervals (CI) following NHANES protocols using the *survey* package in R (v4.1.3) [7]. All statistical significance was assessed at an alpha level of 0.05.

## 3 Results

After weighting, our analytic sample of 2108 adults age ≥ 18 and < 40 years of age represented a population of 88.7 million young adults. We found post‐weighted prevalence estimates of 51.0% (95% CI 48.1–53.9), 32.5% (95% CI 29.9–35.2), 12.7% (95% CI 10.9–14.6), and 3.8% (95% CI 2.4–5.2) for LDL‐C levels of < 100, 100–< 130, 130–< 160 and 160–< 190 mg/dL, respectively. Only 21.5% and 16.3% of young adults with an LDL‐C 130–< 160 and 160–< 190 mg/dL were aware they had elevated cholesterol. Overall, female participants were more prevalent in the lowest category of LDL‐C (< 100 mg/dL), while male participants were more prevalent in all other categories of LDL‐C. The majority of participants self‐identified as Non‐Hispanic White, while participants who self‐identified as Non‐Hispanic Black and Mexican American represented the next two largest demographic groups (Table 1).

**Table 1:** Weighted distribution of non‐LDL‐C cardiovascular disease risk factors by baseline LDL‐C among young adults participating in NHANES 2015 through March 2020.

	< 100 mg/dL	100–< 130 mg/dL	130–< 160 mg/dL	160–< 190 mg/dL
Sample size, unweighted	1053	692	275	88
Sample size, weighted	45 182 038	28 836 836	11 297 812	3 354 802
Gender				
Female	55.3% (50.2, 60.4)	49.1% (44.3, 53.8)	41.2% (33.8, 48.6)	31.0% (17.6, 44.4)
Male	44.7% (39.6, 49.8)	50.9% (46.2, 55.7)	58.8% (51.4, 66.2)	69.0% (55.6, 82.4)
Race and ethnicity				
Non‐Hispanic White	58.3% (51.7, 64.8)	51.6% (44.9, 58.3)	47.3% (39.2, 55.4)	43.0% (27.8, 58.3)
Non‐Hispanic Black	13.2% (9.5, 16.9)	11.4% (7.9, 14.8)	13.6% (8.6, 18.6)	14.1% (7.0, 21.2)
Mexican American	12.4% (7.7, 17.0)	15.7% (11.9, 19.5)	13.8% (7.6, 20.1)	12.6% (3.6, 21.6)
Other Hispanic	7.2% (5.3, 9.1)	8.6% (5.9, 11.3)	8.3% (4.3, 12.4)	10.3% (2.2, 18.4)
Other/Multi‐racial	9.0% (7.1, 10.9)	12.8% (9.3, 16.2)	17.0% (11.7, 22.2)	19.9% (10.2, 29.6)
Family history of ASCVD	8.6% (6.3, 11.5)	9.1% (6.2, 12.6)	7.7% (4.0, 12.8)	9.7% (3.7, 19.5)
High non‐HDL‐C	0.0% (0.0, 0.0)	0.8% (0.2, 2.0)	8.9% (6.5, 11.8)	74.4% (61.6, 84.8)
Metabolic syndrome	15.2% (12.2, 18.5)	26.2% (20.9, 31.9)	31.2% (23.4, 39.7)	41.3% (27.5, 56.1)
Enlarged waist	37.8% (32.5, 43.4)	49.4% (44.0, 54.9)	61.2% (52.4, 69.6)	58.6% (41.3, 74.5)
Hypertension	18.2% (14.9, 21.8)	27.6% (22.7, 32.9)	29.8% (23.0, 37.1)	52.3% (35.8, 68.5)
Low HDL‐C	24.6% (20.8, 28.6)	33.5% (29.0, 38.3)	32.1% (24.8, 40.1)	25.8% (16.3, 37.0)
Elevated glucose	27.2% (23.1, 31.6)	39.8% (34.0, 45.8)	44.2% (35.3, 53.3)	55.7% (43.4, 67.6)
Diabetes mellitus	2.3% (1.4, 3.5)	5.2% (3.6, 7.3)	2.9% (1.3, 5.4)	9.7% (3.7, 19.6)
High triglycerides	5.4% (3.6, 7.6)	8.3% (5.8, 11.3)	18.7% (14.6, 23.3)	20.8% (11.0, 33.6)
CKD	0.0% (0.0, 0.0)	0.0% (0.0, 0.0)	0.0% (0.0, 0.0)	0.0% (0.0, 0.0)
High hs‐CRP	35.1% (30.5, 40.0)	45.8% (39.4, 52.2)	54.9% (45.5, 64.0)	52.9% (43.1, 62.6)
Currently smoking	34.5% (28.8, 40.3)	34.1% (28.8, 39.6)	39.7% (31.9, 47.9)	34.8% (21.7, 49.5)
Ever had blood cholesterol checked	50.9% (46.0, 55.7)	51.9% (45.9, 58.0)	59.0% (49.5, 68.0)	53.4% (35.2, 71.0)
Told by doctor cholesterol was elevated	3.5% (2.3, 4.9)	10.4% (8.0, 13.1)	21.5% (15.5, 28.5)	16.3% (9.2, 25.6)

*Notes:* Numbers in the table are column percentages (95% confidence interval) using weighted estimates. Family history of ASCVD is defined as an affirmation of a father, mother, sister, or brother ever told they had a heart attack or angina < 50 years of age by a medical professions. High non‐HDL‐C is defined as a value ≥ 190 mg/dL. Enlarged waist is defined as greater than 40 inches in males and 35 inches in females. Low HDL‐C is defined as less than 40 mg/dL in males and 50 mg/dL in females. High triglycerides are defined as 175 mg/dL and greater. High hs‐CRP is defined as 2 mg/L or greater.

Abbreviations: ASCVD, atherosclerotic cardiovascular disease; CKD, chronic kidney disease; HDL‐C, high‐density lipoprotein cholesterol; hs‐CRP, high sensitivity C‐reactive protein; non‐HDL‐C, non‐high‐density lipoprotein cholesterol.

Compared to participants with an LDL‐C < 100 mg/dL, those with an LDL‐C 100–< 130, 130–160, and 160–< 190 mg/dL were more likely to have metabolic syndrome, an enlarged waist, hypertension, low HDL‐C, elevated glucose, diabetes mellitus, high triglycerides, and high hs‐CRP (Table 2). Due to insufficient sample size of individuals with high non‐HDL‐C and CKD within each category of LDL‐C, valid association estimates could not be determined.

**Table 2:** Odds ratios (95% Confidence Intervals) of non‐LDL‐C cardiovascular risk factors by baseline LDL‐C among young adults participating in NHANES 2015 through March 2020.

	< 100 mg/dL	100–< 130 mg/dL	130–< 160 mg/dL	160–< 190 mg/dL
Family history of ASCVD	1 (reference)	1.06 (1.04, 1.08)	0.88 (0.86, 0.90)	1.14 (1.12, 1.17)
Metabolic syndrome	1 (reference)	1.98 (1.96, 2.00)	2.54 (2.49, 2.58)	3.94 (3.87, 4.02)
Enlarged waist	1 (reference)	1.61 (1.59, 1.63)	2.59 (2.54, 2.64)	2.33 (2.26, 2.39)
Hypertension	1 (reference)	1.72 (1.69, 1.74)	1.91 (1.88, 1.93)	4.94 (4.83, 5.05)
Low HDL‐C	1 (reference)	1.55 (1.54, 1.56)	1.45 (1.43, 1.48)	1.07 (1.05, 1.08)
Elevated glucose	1 (reference)	1.77 (1.74, 1.79)	2.12 (2.08, 2.15)	3.37 (3.32, 3.41)
Diabetes mellitus	1 (reference)	2.38 (2.35, 2.41)	1.28 (1.26, 1.31)	4.64 (4.53, 4.74)
High triglycerides	1 (reference)	1.59 (1.57, 1.62)	4.05 (3.97, 4.10)	4.61 (4.51, 4.71)
High hs‐CRP	1 (reference)	1.56 (1.54, 1.58)	2.24 (2.21, 2.28)	2.08 (2.05, 2.10)
Currently smoking	1 (reference)	0.98 (0.97, 1.00)	1.25 (1.24, 1.27)	1.02 (1.00, 1.04)

*Notes:* Unadjusted odds ratios presented. Family history of ASCVD is defined as an affirmation of a father, mother, sister, or brother ever told they had a heart attack or angina < 50 years of age by a medical professions. Enlarged waist is defined as greater than 40 inches in males and 35 inches in females. Low HDL‐C is defined as less than 40 mg/dL in males and 50 mg/dL in females. High triglycerides are defined as 175 mg/dL and greater. High hs‐CRP is defined as 2 mg/L or greater.

Abbreviations: ASCVD, atherosclerotic cardiovascular disease; CKD, chronic kidney disease; HDL‐C, high‐density lipoprotein cholesterol; hs‐CRP, high sensitivity C‐reactive protein; non‐HDL‐C, non‐high‐density lipoprotein cholesterol.

**Figure 1:**
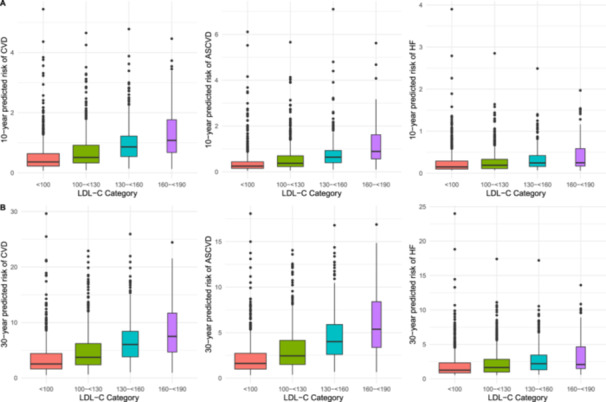
Distribution of predicted risk of CVD, ASCVD, and HF by the PREVENT Equation for young adults by baseline LDL‐C among young adults participating in NHANES 2015 through March 2020. (A) The 10‐year predicted risk of CVD, ASCVD, and HF by LDL‐C and (B) the 30‐year predicted risk of CVD, ASCVD, and HF by LDL‐C. All units of LDL‐C are mg/dL.

Overall, the median 10‐year risk of incident CVD, ASCVD, or HF was less than 1% for all categories of baseline LDL‐C, except for CVD risk among participants with an LDL‐C 160‐ < 190 mg/dL which was 1.1% (IQR 0.7–1.8; Figure 1A). When using the 30‐year time horizon to predict risk using the PREVENT equation, the median risk of incident CVD exceeded 5% only in the 130–< 160 and 160–< 190 mg/dL LDL‐C categories (6.0%, IQR 3.8%–8.4% and 7.5%, IQR 4.7–11.7, respectively; Figure 1B). There was nearly a threefold increase in median 30‐year CVD risk between young adults with an LDL‐C < 100 mg/dL (2.5%, IQR 1.6–4.4) compared to young adults with an LDL‐C 160–< 190 mg/dL (7.6%, IQR 4.7–11.7). The median 30‐year predicted risk of ASCVD only exceeded 5% in the 160–< 190 mg/dL LDL‐C category with regard to incident ASCVD (5.4%, IQR 3.4–8.4). The median 30‐year predicted risk of HF was low among all categories (Table 3). The distribution of risk for CVD, ASCVD, and HF is visualized over a 10‐year time horizon and 30‐year time horizon in Figure 1A,B, respectively.

**Table 3:** Predicted 10‐ and 30‐year median CVD, ASCVD, and HF risk by LDL‐C category based on the PREVENT equation for young adults participating in NHANES 2015 through March 2020.

	< 100 mg/dL	100–< 130 mg/dL	130–< 160 mg/dL	160–< 190 mg/dL
*Risk, median (interquartile range)*				
Current age^a^				
10‐year risk				
CVD	0.4% (0.2, 0.6)	0.5% (0.3, 0.9)	0.9% (0.5, 1.22)	1.1% (0.7, 1.8)
ASCVD	0.3% (0.2, 0.4)	0.4% (0.2, 0.7)	0.6% (0.4, 0.9)	0.8% (0.5, 1.3)
HF	0.2% (0.1, 0.3)	0.2% (0.1, 0.4)	0.3% (0.2, 0.5)	0.3% (0.2, 0.8)
30‐year risk				
CVD	2.5% (1.6, 4.4)	3.7% (2.4, 6.2)	6.0% (3.8, 8.4)	7.5% (4.7, 11.7)
ASCVD	1.6% (1.0, 2.8)	2.5% (1.5, 4.2)	4.0% (2.6, 5.9)	5.4% (3.4, 8.4)
HF	1.3% (0.9, 2.3)	1.6% (1.0, 2.8)	2.2% (1.3, 3.5)	2.1% (1.5, 4.6)

Abbreviations: ASCVD, atherosclerotic cardiovascular disease; CVD, cardiovascular disease; HF, heart failure; LDL‐C, low‐density lipoprotein cholesterol; PREVENT, Predicting Risk of cardiovascular disease EVENTs.

^a^ For adults < 30 years of age, a standardized age of 30 years was used for the calculation.

The unadjusted association between higher LDL‐C and 30‐year predicted risk of CVD and ASCVD was statistically significant (OR 1.11, 95% CI 1.09–1.13 and OR 1.13, 95% CI 1.11–1.15). After adjustment, the association between higher LDL‐C and 30‐year predicted risk of CVD and ASCVD was attenuated though remained statistically significant (OR 1.05, 95% CI 1.03–1.07 and OR 1.06, 95% CI 1.04–1.08). There was no statistically significant interaction between LDL‐C and number of non‐LDL‐C risk factors with a 30‐year predicted risk of CVD or ASCVD (*p* = 0.43 and *p* = 0.46, respectively; Table 4).

**Table 4:** Association between LDL‐C, cumulative non‐LDL‐C risk factors, and the interaction between LDL‐C and cumulative non‐LDL‐C risk factors with a 30‐year predicted risk of CVD, ASCVD, and HF by PREVENT among young adults participating in NHANES 2015 through March 2020.

	Beta coefficient (SE)	OR (95% CI)	*p* value
Cardiovascular disease			
Model 1			
LDL‐C^a^	0.11 (0.01)	1.11 (1.09–1.13)	< 0.001
Model 2			
LDL‐C^a^	0.04 (0.01)	1.04 (1.03–1.06)	< 0.001
Cumulative non‐LDL‐C risk factors	0.14 (0.01)	1.15 (1.14–1.16)	< 0.001
Model 3			
LDL‐C^a^	0.05 (0.01)	1.05 (1.03–1.07)	< 0.001
Cumulative non‐LDL‐C risk factors	0.16 (0.02)	1.17 (1.12–1.23)	< 0.001
LDL‐C*cumulative non‐LDL‐C risk factors	−0.01 (0.01)	1.00 (0.99–1.00)	0.43
Atherosclerotic cardiovascular disease			
Model 1			
LDL‐C^a^	0.12 (0.01)	1.13 (1.11–1.15)	< 0.001
Model 2			
LDL‐C^a^	0.05 (0.01)	1.06 (1.04–1.07)	< 0.001
Cumulative non‐LDL‐C risk factors	0.14 (0.01)	1.15 (1.14–1.17)	< 0.001
Model 3			
LDL‐C^a^	0.06 (0.01)	1.06 (1.04–1.08)	< 0.001
Cumulative non‐LDL‐C risk factors	0.16 (0.02)	1.17 (1.12–1.22)	< 0.001
LDL‐C*cumulative non‐LDL‐C risk factors	−0.01 (0.01)	1.00 (0.99–1.00)	0.46
Heart failure			
Model 1			
LDL‐C^a^	0.03 (0.08)	1.03 (0.88–1.21)	0.73
Model 2			
LDL‐C^a^	b	b	b
Cumulative non‐LDL‐C risk factors	b	b	b
Model 3			
LDL‐C^a^	b	b	b
Cumulative non‐LDL‐C risk factors	b	b	b
LDL‐C*cumulative non‐LDL‐C risk factors	b	b	b

*Notes:* Model 1: Unadjusted association between 30‐year predicted risk of CVD, ASCVD, and HF, separately, and LDL‐C. Model 2: Adjusted for age, gender, race, LDL‐C, and cumulative non‐LDL‐C risk factors (metabolic syndrome, enlarged waist, hypertension, elevated triglycerides, low HDL‐C, elevated glucose, elevated hs‐CRP). Model 3: Adjusted for all variables in Model 2 plus LDL‐C*cumulative non‐LDL‐C risk factors.

Abbreviations: CI, confidence interval; LDL‐C, low‐density lipoprotein cholesterol; OR, odds ratio; SE, standard error.

^a^ Per 10 mg/dL increase in LDL‐C.

^b^ Results not reported due to numerical instability given low predicted values.

## 4 Discussion

In this study, young adults exposed to excess CVD risk from atherogenic lipids—which are readily modifiable—faced a greater prevalence of non‐LDL‐C risk factors—not all of which are captured in the calculation of predicted 10‐ and 30‐year CVD event risk. Moreover, both LDL‐C and non‐LDL‐C CVD risk factors were independently associated with 30‐year predicted CVD risk. While it may be expected that young adults with greater LDL‐C will have greater non‐LDL‐C CVD risk factors, the current analysis provides an estimation of this burden in a modern cohort of young adults and demonstrates the strength of association with predicted risk of CVD.

In the current treatment paradigm, young adults with excess atherogenic lipids experience longer time‐exposure to a pro‐atherogenic environment. This may be particularly problematic as our study highlights how young adults with the highest LDL‐C also have the highest prevalence of non‐LDL‐C risk factors, which are subject to further increase in burden over time [12, 13, 14, 15]. Results from our analysis, in concert with prior studies, demonstrate that cardiovascular risk does not begin in middle age—young adults exposed to LDL‐C and non‐LDL‐C risk factors have higher predicted long‐term cardiovascular risk.

Reassuringly, and as expected, we did find that the median predicted 10‐ and 30‐year risk of CVD, ASCVD, and HF remained relatively low for all young adults. However, the predicted risk is likely to be a conservative estimate due to the assumption that CVD risk factor levels remain static. Among young adults with an LDL‐C ≥ 130 mg/dL, there was a non‐negligible 6‐7% median predicted risk of CVD over 30 years, and these individuals also had the greatest prevalence of non‐LDL‐C risk factors. Although the PREVENT equation tends to predict a lower risk than the PCE, current risk thresholds are based on PCE‐predicted risk, which may lead to fewer statin recommendations when using PREVENT‐predicted risk with current guideline thresholds [16]. However, the PCE is not validated in young adults or over a 30‐year time horizon [5, 17]. New guidelines will need to incorporate new thresholds and age considerations for statin initiation [17].

Previous studies using trial data showed a 19%–22% reduction of ASCVD risk per 38 mg/dL reduction in LDL‐C, and this benefit has been demonstrated across a range of baseline risk levels, which suggests that high‐risk young adults might benefit from early initiation of LLT [18, 19, 20]. Lowering LDL‐C levels earlier may even supersede the benefit found among older adults; data from the Coronary Artery Risk Development in Young Adults (CARDIA) study demonstrated an increased risk based on exposure time of LDL‐C, with exposure to elevated LDL‐C from age 18 to 30 years posing greater risk than later exposure [3]. Further, nationally representative modeling data have shown that initiation of statin therapy among young adults 18–39 with an LDL‐C ≥ 130 mg/dL can significantly reduce ASCVD and improve quality‐adjusted life years more than what lifestyle interventions can achieve alone [21].

While we found that young adults with higher levels of LDL‐C concomitantly have a higher prevalence of non‐LDL‐C risk factors, the clinical approach to managing LDL‐C and non‐LDL‐C risk factors is not harmonious. Non‐atherogenic lipid risk factors, such as hypertension, diabetes, or obesity, are conceptualized and routinely treated in young adults. In contrast, elevated LDL‐C in young adults is often ignored until atherosclerosis is present later in life. Importantly, coronary artery disease (CAD) requires atherogenic lipids to become trapped and modified within the endothelial layer for clinically significant pathology to develop. Mechanistically, atherogenic lipids, most notably LDL‐C, are required for CAD development, though they may not be sufficient by themselves as other risk factors (e.g., endothelial dysfunction and inflammation) often propagate CAD progression [22, 23]. The magnitude of impact of non‐LDL‐C risk factors, such as inflammation or elevated glucose, on the development of overt CVD can vary individually. Although we found no interaction between LDL‐C and non‐LDL‐C risk factors in predicting event risk, it is likely that the individual composition of non‐LDL‐C risk factors would influence non‐LLT therapeutic focus. Nonetheless, atherogenic lipids remain necessary for CAD and often remain untreated. As a result of delayed intervention, young adults unnecessarily progress to higher‐risk states despite the availability of safe, effective, and low‐cost LLT options [24, 25, 26, 27]. To fully achieve optimal cardiovascular risk control among young adults, guidance is needed for each causal determinant of CVD, including elevated LDL‐C.

Lifestyle interventions, which are the first‐line intervention to reduce cardiovascular risk, are beneficial, but often insufficient. The limitation of lifestyle interventions is in part due to the significant burden that can be required to change factors such as diet and exercise, particularly among disadvantaged young adults who may have less financial resources and access to healthcare compared to older adults [28, 29]. Though there is apprehension about long‐term LLT use in young adults, there is robust evidence of the safety, effectiveness, and tolerability for long‐term statin use, as evidence by pediatric patients with familial hypercholesterolemia [30, 31]. Ten‐year risk estimation may be of little use to young adults (i.e., < 40 years of age) who have a low likelihood of near‐term event, but high long‐term risk [32]. However, the PREVENT calculator was released which allows for cardiovascular risk to be calculated for adults as young as 30 years of age [5, 6]. Further guidance is needed on how to integrate the 30‐year CVD risk horizons into clinical decisions to utilize pharmacotherapy in concert with non‐pharmacotherapy interventions, especially for younger adults. While the advent of the PREVENT equation may help to identify young adults at high risk for CVD and determine the absolute risk reduction through early intervention, there remains little guidance on how we consider incorporating such a tool in the management of atherogenic lipids in young adults [5, 6]. It is possible that relying exclusively on risk scores for guiding treatment in young adults, as we do for many older adults, may not be appropriate. For example, in the YOUNG‐MI registry, over 50% of adults with a myocardial infarction before the age of 50 would not have been recommended statin therapy based on current risk‐based thresholds [32, 33]. Future trials may help inform how to approach LLT among young adults. The Prevent Coronary Artery Disease (PRECAD), a trial designed to reduce LDL‐C < 70 mg/dL among young adults who maintain optimal blood pressure and glucose may provide insight into the impact of early LDL‐C reduction with pharmacotherapy and subclinical atherosclerosis [34]. The results of PRECAD will hopefully offer data to further guide clinical recommendations for when, and if, pharmacotherapy may be beneficial for young adults. While we currently lack analogous clinic trial data for young adults to those for adults in mid‐ and late‐life, future work to understand the long‐term clinical implications of targeting LDL‐C among high‐risk young adults, such as in the PRECAD trial, would be logically cogent with how we handle other risk factors for CVD in younger adults and may complement a holistic approach to cardiovascular risk reduction.

There are notable strengths to the current study. First, NHANES is a nationally representative sample and provides reliable and updated representation across demographics. Moreover, the measurement of exam metrics, such as BP, and laboratory measures, such as LDL‐C, are protocolized and, as such, have little variation from sample to sample. Limitations include the observational nature of our analysis, which risks being subject to unmeasured confounding. Our study is additionally limited by the lack of clinical events and a prospective design which would allow for a sufficient time horizon to capture these events so as to determine the magnitude effect of LDL‐C and non‐LDL‐C risk factors. Additionally, the median age of our study population is 28 years and thus risk estimates for those we imputed an age of 30 for to calculate 10‐ and 30‐year risk of CVD, ASCVD, and HF are prone to underestimate risk given that risk factors tend to accumulate over time. Furthermore, we cannot comment on if reducing LDL‐C would improve long term cardiovascular outcomes, though this study adds exploratory epidemiologic evidence suggesting LLT in high‐risk young adults could be a fruitful area for future research. Finally, very few younger adults had certain non‐LDL‐C risk factors (e.g., CKD), limiting our ability to comment on their association with LDL‐C.

## 5 Conclusion

The prevalence of non‐LDL‐C CVD risk factors is common among young adults and most common among those with elevated LDL‐C. The progression from risk, to atherosclerosis, to clinical CVD results from multiple pathways, including from the presence of atherogenic lipids. Bending the curve away from ASCVD later in life requires greater investment in understanding the impact of prolonged exposure to causal determinants of CVD beginning early in life and when intervention is warranted among high‐risk young adults. Eventually, intervention studies may be warranted to determine if earlier LLT initiation in high‐risk young adults may help mitigate long‐term risk before atherogenic deposition begins.

## Conflicts of Interest

The authors declare no conflicts of interest.

## Data Availability

Our Expects Data Policy requires a Data Availability Statement, even if no data are available, so please enter one in the space below. Sample statements can be found here. Please note that this statement will be published alongside your manuscript, if it is accepted for publication. The data that support the findings of this study are available in NHANES at https://wwwn.cdc.gov/Nchs/Nhanes/. All data in the current manuscript is publicly available at: https://www.cdc.gov/nchs/nhanes/index.htm. These data were derived from the following resources available in the public domain: NHANES, https://www.cdc.gov/Nchs/Nhanes/.
